# SNBRFinder: A Sequence-Based Hybrid Algorithm for Enhanced Prediction of Nucleic Acid-Binding Residues

**DOI:** 10.1371/journal.pone.0133260

**Published:** 2015-07-15

**Authors:** Xiaoxia Yang, Jia Wang, Jun Sun, Rong Liu

**Affiliations:** Agricultural Bioinformatics Key Laboratory of Hubei Province, College of Informatics, Huazhong Agricultural University, Wuhan, Hubei, People’s Republic of China; The University of North Carolina at Charlotte, UNITED STATES

## Abstract

Protein-nucleic acid interactions are central to various fundamental biological processes. Automated methods capable of reliably identifying DNA- and RNA-binding residues in protein sequence are assuming ever-increasing importance. The majority of current algorithms rely on feature-based prediction, but their accuracy remains to be further improved. Here we propose a sequence-based hybrid algorithm SNBRFinder (Sequence-based Nucleic acid-Binding Residue Finder) by merging a feature predictor SNBRFinder^F^ and a template predictor SNBRFinder^T^. SNBRFinder^F^ was established using the support vector machine whose inputs include sequence profile and other complementary sequence descriptors, while SNBRFinder^T^ was implemented with the sequence alignment algorithm based on profile hidden Markov models to capture the weakly homologous template of query sequence. Experimental results show that SNBRFinder^F^ was clearly superior to the commonly used sequence profile-based predictor and SNBRFinder^T^ can achieve comparable performance to the structure-based template methods. Leveraging the complementary relationship between these two predictors, SNBRFinder reasonably improved the performance of both DNA- and RNA-binding residue predictions. More importantly, the sequence-based hybrid prediction reached competitive performance relative to our previous structure-based counterpart. Our extensive and stringent comparisons show that SNBRFinder has obvious advantages over the existing sequence-based prediction algorithms. The value of our algorithm is highlighted by establishing an easy-to-use web server that is freely accessible at http://ibi.hzau.edu.cn/SNBRFinder.

## Introduction

Protein-nucleic acid interactions are central to various fundamental biological processes, especially those related to replication, transcription, and translation [1, 2]. Although the advanced experimental techniques have accelerated our understanding of the mechanism of recognition and binding between protein and DNA/RNA in the past twenty years, the principles governing protein-nucleic acid interactions remain to be further exploited. Computational prediction of DNA- and RNA-binding residues might offer new insights into the elucidation of this problem.

As the number of experimentally solved protein-nucleic acid complexes increases steadily, a variety of structure-based algorithms have been proposed to predict DNA- or RNA-binding residues. These approaches can roughly be partitioned into three classes: (i) the feature-based class; (ii) the template-based class; (iii) the hybrid class. As for the first class, the predictors use various structural features of the target residues in conjunction with their local contexts as the inputs of different machine learning techniques or customized scoring functions to predict their binding function [3–16]. As for the second class, the predictors utilize structure alignment algorithm as searching engine to retrieve the reliable template of query protein and recognize the binding residues with higher confidence based on the simulated complex structure [17–20]. Unfortunately, not all the proteins can achieve reasonable templates to identity their binding residues. In these cases, the feature-based predictor might complement the shortcoming of the template-based predictor. Based on this assumption, we recently built two hybrid predictors DNABind and RBRDetector [21, 22], belonging to the third class, which respectively improved DNA- and RNA-binding residue predictions by leveraging the complementary nature of feature- and template-based approaches. Nevertheless, the major limitation of structure-based algorithms is that they can only be applicable to a relatively small range of proteins whose structures are available. Due to the fact that the number of solved structures substantially lags behind that of protein sequences, it is therefore more urgent to develop effective and efficient computational tools for annotating nucleic acid-binding residues from protein sequence.

Theoretically, the sequence-based approaches could also be divided into the three classes similar to those of the structure-based counterparts. In particular, we expect that the integrative prediction strategy can be extended to sequence-based prediction, which might have a broader application in the real situation. However, the majority of current sequence predictors belong to the first class [23–33]. To our knowledge, only the HomPRIP and RNABindRPlus algorithms proposed by Wilia et al. [34] respectively utilize the template-based and hybrid strategies to recognize RNA-binding residues in protein sequence. HomPRIP uses the sequence alignment program BLAST [35] to retrieve the templates of query sequence with a relaxed sequence identity constraint (95%), while RNABindRPlus is an integrative predictor that uses logistic regression to merge the predictions from HomPRIP and another feature-based predictor named SVMOpt. Wilia et al. [34] show that HomPRIP achieved promising performance for the query sequences whose close homologs can be found and RNABindRPlus was superior to SVMOpt due to the incorporation of HomPRIP. Despite their pioneering work, the methodology might be further improved from the following aspects. First, the template detection based on protein sequence is actually the fold recognition problem. Zhao et al. [36, 37] recently exploited two profile alignment tools SPARKS-X and HHblits, which showed better performance than BLAST, to respectively distinguish RNA- and DNA-binding proteins from nonbinding proteins. Intuitively, the profile alignment algorithms could be more sensitive to finding the templates (especially the remote ones) of nucleic acid-binding sequences, which is definitely helpful for enhancing template-based prediction. Second, the relaxed sequence identity threshold used to remove homologs would result in overestimation of the template and hybrid predictors. More stringent constraint such as the 30% cutoff should be applied to performance evaluation, which can objectively reflect the usefulness and applicable range of the proposed methods. Third, the feature-based module SVMOpt only uses sequence profile to characterize each residue and disregards other residue properties related to protein-nucleic acid interactions. Additional sequence descriptors that are complementary to sequence profile should be exploited for improving feature-based prediction. Fourth, if the proposed methods work well for RNA-binding residue prediction, one might expect that the methodology can also be applicable to DNA-binding residue prediction due to the similarities between them.

With this background, we attempted to develop a sequence-based hybrid algorithm for predicting nucleic acid-binding residues. To this end, using support vector machine algorithm, we implemented a feature-based predictor termed SNBRFinder^F^ whose inputs are composed of comprehensive sequence-derived descriptors. Meanwhile, we built a template-based predictor termed SNBRFinder^T^ in which the profile hidden Markov models were used to retrieve the optimal template of the query sequence. The extensive and stringent evaluation reveals that SNBRFinder^F^ was superior to the sequence profile-based predictor, and SNBRFinder^T^ yielded comparable performance to the template-based predictor established using structural alignment. By combining the individual methods, the hybrid predictor termed SNBRFinder remarkably improved the performance for both DNA- and RNA-binding residue predictions. Finally, we demonstrate that SNBRFinder not only got comparable or superior performance in comparison with RNABindRPlus, but also outperformed the existing sequence-based feature predictors. This work suggests that our proposed methodology could be further applicable for annotating other functional residues on a genome scale.

## Materials and Methods

### Datasets

#### DB312 and RB264

DB312 is a DNA-binding protein dataset including 312 chains, while RB264 is an RNA-binding protein dataset composed of 264 chains. These two datasets were generated in our earlier works [21, 22]. All the entries in DB312 (or BR264) satisfy the following constraints: (i) the chain is extracted from the complex structure solved by X-ray crystallography with a resolution better than 3Å; (ii) the chain is not shorter than 40 amino acids long and includes at least five DNA-binding (or RNA-binding) residues; (iii) no two chains share more than 30% sequence identity. Here a residue was considered as a binding residue if it contains at least one heavy atom within 4.5Å of any atom in the bound DNA (or RNA) molecule. Based on this definition, the DB312/RB264 dataset contains 8596/8995 binding residues and 65162/58541 nonbinding residues. We utilized these two datasets for cross-validation experiments and for training the final prediction model in our web server.

#### DB123, DB232, DB374, RB106, RB144, and RB198

DB123, DB232, and DB374 are three DNA-binding protein datasets including 123, 232, and 374 chains respectively, and RB106, RB144, and RB198 are three RNA-binding protein datasets including 106, 144, and 198 chains respectively. These six datasets were established by the existing studies [21, 38–40]. The maximum pairwise sequence identity of DB123 and DB374 is 25%, while the corresponding measure of the remaining datasets is 30%. We also performed cross-validation on these datasets for demonstrating the robustness of our proposed algorithm. The binding residues in each dataset were defined according to the original criterion in the references. More details about these datasets are provided in S1 Table.

#### DB35 and RB36

In addition to sequence-based prediction, the structural model of a given sequence can also be used to infer its binding regions. Dror et al. [41] collected 35 DNA-binding proteins (DB35) and 36 RNA-binding proteins (RB36), where the chains share less than 25% sequence identity. They produced the structural model of each chain by running the I-TASSER program [42] with the 95% sequence identity cutoff. For these datasets, the binding residues were generated using the same definition of DB312 and RB264. DB35 and RB36 were used as independent datasets to estimate our sequence-based method, the performance of which was to be compared with that of structural model-based prediction.

#### DB33, RB49, and RB44

DB33, RB49, and RB44 are three well-established nucleic acid-binding protein datasets, which include 33, 49, and 44 chains, respectively. DB33 and RB49 were built by Nagarajan et al. [43, 44] (the Dataset2 in their works) and RB44 was established by Puton et al. [45]. The pairwise sequence identity of DB33 and RB49 is less than 30%, while this measure of RB44 is not greater than 40%. Since these datasets have been used to compare the existing approaches, we also considered them as independent datasets to estimate our method. To make a direct comparison, we used the original criterion to define the binding residues in each dataset.

## Sequence-Based Residue Representation

In the current work, we extracted various sequence signatures, such as sequence profile, residue conservation scores, predicted structural features, physicochemical properties, interface propensity, sequential position, and global features, to quantitatively characterize each residue in a query sequence. Details about these features are given in the following sections.

### Sequence profile

Sequence profile is an effective indicator for measuring the evolutionary conservation of each residue in a protein sequence. The position specific scoring matrix (PSSM) of each query sequence was generated by conducting PSI-BLAST searches [46] against NCBI non-redundant database with three iterations and the E-value threshold of 0.001. The elements of PSSM were normalized using the standard logistic function as:

$$f(x)=\frac{1}{1+e^{-x}}$$

where *x* is the raw element value of the PSSM.

### Residue conservation scores

Besides sequence profile, the conservation of each residue was represented by the other three features, such as Shannon entropy (SE) [47], relative entropy (RE) [48], and Jensen-Shannon divergence score (JSD) [49]. They were calculated using the weighted observed percentages derived from the multiple sequence alignment (MSA) generated by PSI-BLAST. Especially, the RE and JSD measures further consider the background frequency of amino acids. The definition of these features is given as following:

$$SE=-\sum_{\alpha \in AA}p_{i}(\alpha )\log p_{i}(\alpha )$$

$$RE=\sum_{\alpha \in AA}p_{i}(\alpha )\log\frac{p_{i}(\alpha )}{q(\alpha )}$$

$$JSD=\lambda \sum_{\alpha \in AA}p_{i}(\alpha )\log\frac{p_{i}(\alpha )}{c_{i}(\alpha )}+(1-\lambda )\sum_{\alpha \in AA}q(\alpha )\log\frac{q(\alpha )}{c_{i}(\alpha )}$$

where *p*_*i*_(*α*) denotes the weighted occurring frequency of residue type *α* in the *i* ^th^ column of the MSA, *q*(*α*) denotes the background frequency that was derived from the overall amino acid distribution in the BLOSUM62 alignments, *c*_*i*_(*α*) is a combined frequency defined as *c*_*i*_(*α*) = λ*p*_*i*_(*α*) + (1- λ) *q*(*α*), and λ is a prior weight and is set to 0.5.

### Predicted structural features

Predicted structural features could provide valuable information as the native structure of the query protein is not available. For each residue, the predicted secondary structure, solvent accessibility, and backbone dihedral angles were generated using the SPINEX software [50], while the predicted disorder score was produced by the DISOPRED program [51]. The predicted solvent accessibility should be normalized by the maximum surface area [52], and the predicted dihedral angles should be divided by 180.

### Physicochemical properties

Physicochemical attributes of an amino acid might play important roles in protein-nucleic acid interactions. To capture the specific properties of each residue type, we chose five amino acid indices from the AAindex database [53], including hydrophobicity, hydrophilicity, number of electrostatic charge, number of potential hydrogen bonds, and isoelectric points (S2 Table). These attribute values were to be normalized using the standard logistic function.

### Interface propensity

Interface propensity measures the relative importance of different amino acids in nucleic acid-binding interface. We calculated the interface propensity of each residue type which is defined as the ratio of the amino acid frequency in the binding interface to that in the whole protein sequence. We then normalized the raw values using the following equation:

$$P_{i}^{norm}=\frac{P_{i}\;\text{-}\;P_{min}}{P_{max}\;\text{-}\;P_{min}}$$

where *P*_*i*_ denotes the propensity of residue type *i*, and *P*_*max*_ and *P*_*min*_ denote the maximum and minimum values among all the residue types, respectively.

### Sequential position

Sequential position represents the relative location of each residue in a whole sequence. Two features proposed by Chen et al. [54], termed as terminus indicator and secondary structure segment indicators, were calculated in this study. For each residue, the terminus indicator was assigned to 1 if this residue is located in the first or the last three positions in a sequence, otherwise it was set to 0. The second measure, including the helix, strand, and coil indicators, is used to annotate whether a helix/strand segment predicted by SPINEX was observed in the flanking regions of the target residue. We assigned the helix/strand indicator of each residue as 1 if there exists a helix/strand segment in its neighborhood, and the coil indicator is thus set to 0. More details can be found in Chen et al.'s paper [54].

### Global features

Global information of a query sequence was represented by two features in this work. One is the sequence length and the other is the global amino acid composition of each protein sequence. Note that the sequence length was to be divided by 1000 for normalization.

### SNBRFinderF: A Sequence-Driven Feature Predictor

In this work, we established a feature-based predictor termed SNBRFinder^F^ for identifying DNA- or RNA-binding residues in a query sequence. We selected the support vector machine (SVM) algorithm to establish the prediction model. A sliding window composed of the target residue and its five flanking residues on each side was utilized to represent its sequential microenvironment. The window size was chosen based on our earlier studies. All the features mentioned in the previous section were used as the inputs of the SVM algorithm. The LIBSVM package [55] was utilized to implement this predictor and the radial basis function was selected as the kernel. A probability score was assigned to each residue by LIBSVM. By conducting a grid search, we find that as the values of C and γ were set to 10 and 0.1 in the kernel function, our predictor can achieve optimal performance for both DNA- and RNA-binding residues.

### SNBRFinderT: A Sequence-Driven Template Predictor

Besides SNBRFinder^F^, we developed an alignment-based method termed SNBRFinder^T^ to recognize putative binding residues only using sequence information, in which the HHblits program [56] was utilized to implement the prediction model. HHblits is a recently published fold recognition algorithm that represents both query and database sequences by profile hidden Markov models (HMMs). In our study, the query and template sequences were transformed into the HMMs by searching them against the Uniprot20 database where the pairwise sequence identity is around 20%. The query HMM was then compared with each HMM in the template library. Default parameters were utilized in the HHblits program. For each query and template pair, HHblits outputs a probability score, ranging from 0 to 100%, for evaluating the similarity between the aligned HMMs. Based on this measure, the top-ranked template was chosen to accomplish the alignment-based prediction. Concretely, one residue in the query sequence was predicted to be a binding residue with a probability score of 1 if this residue was matched with a binding residue in the optimal template, otherwise with a probability score of 0.

### SNBRFinder: A Sequence-Driven Hybrid Predictor

**Fig 1:**
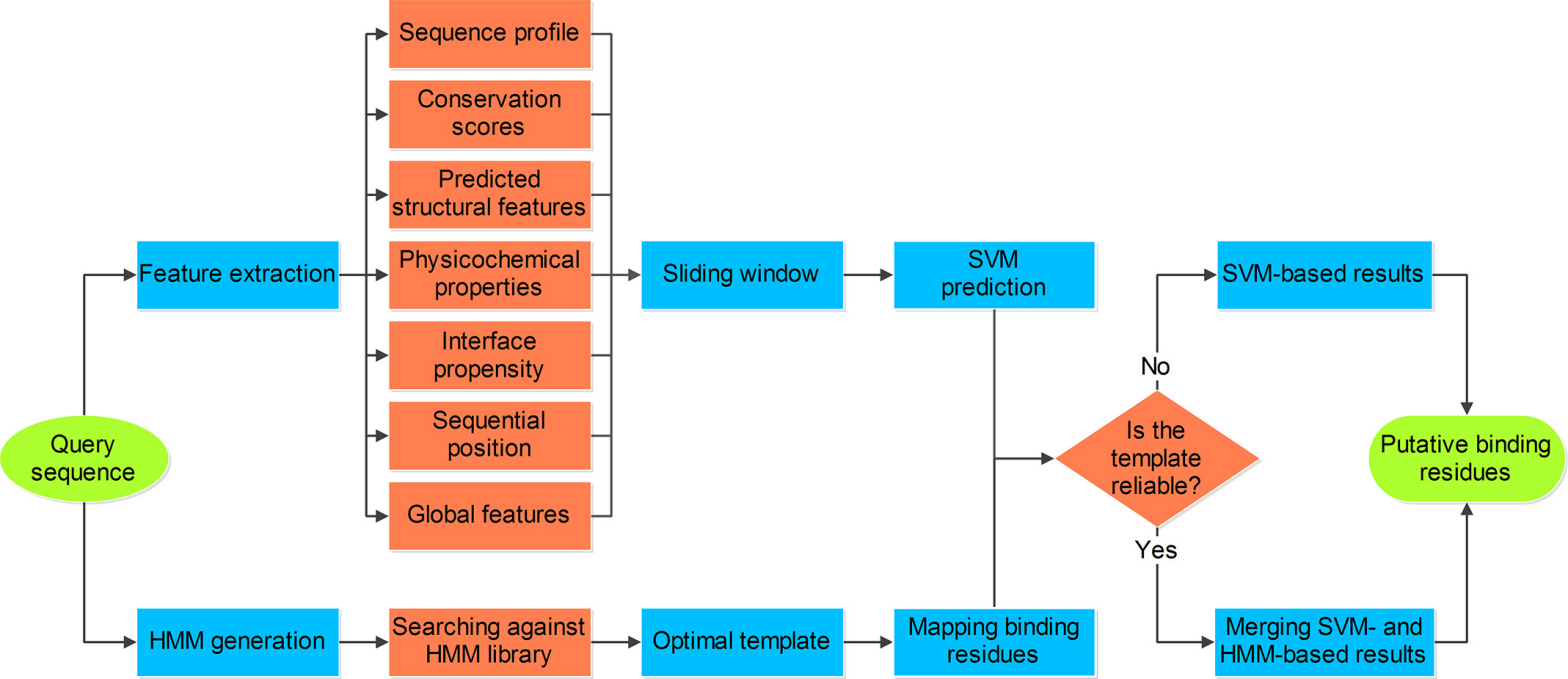
Flowchart of our SNBRFinder algorithm. SNBRFinder is a sequence-based hybrid prediction algotirhm comprising a feature-based predictor SNBRFinder^F^ and a template-based predictor SNBRFinder^T^. SNBRFinder^F^ was built using the support vector machine algorithm whose inputs include comprehensive sequence descriptors and SNBRFinder^T^ was implemented with the sequence alignment algorithm based on profile hidden Markov models.

The current work is motivated by our previous findings that both DNA- and RNA-binding residue predictions can be remarkably improved by merging the structure-based feature and template approaches [21, 22]. Here this strategy was extended to develop a sequence-driven integrative algorithm, referred to as SNBRFinder, for identifying nucleic acid-binding residues. As illustrated in Fig 1, if the query sequence can find a reasonable template using HHblits, we considered the linear combination of the outputs from the feature and template predictors as the final result. Otherwise, the prediction result was individually dependent on the output from the feature predictor. We calculated the final probability score of each residue as following:

$$\left\{ \begin{array}{l} Cscore=\alpha Fscore+(1\;\text{-}\;\alpha )Tscore\;if\;HHscore\geq cutoff\\ Cscore=Fscore\;Otherwise \end{array}\right.$$

where *HHscore* denotes the similarity score between the query sequence and its best template, *Fscore* and *Tscore* represent the outputs of SNBRFinder^F^ and SNBRFinder^T^, respectively. By thoroughly testing the parameters, we find that as the values of *α* and *cutoff* were assigned to 0.6 and 85%, SNBRFinder attained optimal performance for both types of binding residues.

### Test Procedure and Evaluation Measures

In this study, we utilized cross-validation and independent test to evaluate the proposed predictors. We first performed 5-fold cross-validation on the DB312 and RB264 datasets, respectively. In this procedure, we randomly divided DB312/RB264 into five partitions. For each run, four subsets were merged to construct the training set and template library, and the rest one was considered as the test set. Unlike our previous work, here we used all the negative samples (nonbinding residues) to train the machine learning model, because this modification resulted in superior prediction performance (shown later). Similarly, we checked our predictors using different datasets collected in the existing studies (e.g., DB123, DB232, DB374, RB106, RB144, and RB198) by 5-fold cross-validation. In addition, we considered DB312/RB264 as the training set and template library, and performed independent test on the remaining datasets used in this work (e.g., DB35, RB36, DB33, RB49, and RB44). To avoid the bias induced by close homologs, we removed the chains in DB312/RB264 sharing greater than 30% sequence identity with any chain in the independent dataset. The prediction results were to be estimated in terms of two different ways, including residue- and chain-based evaluations, the details of which can be found in Walia et al.'s paper [39]. In agreement with our previous work, the commonly used evaluation measures, such as recall, precision, F1, accuracy (ACC), Matthews correlation coefficient (MCC), and the area under the receiver operating characteristic curve (AUC), were applied to performance assessment.

### Statistical Analysis

Statistical tests were conducted to evaluate the significance of performance difference between a given pair of predictors. We calculated the MCC value of each chain for different predictors and compared the corresponding paired MCC values. If these values come from a normal distribution, as suggested by the Shapiro-Wilk test, we utilized the paired *t*-test to evaluate significance. Otherwise, we used the Wilcoxon signed-rank test. As the *P*-value<0.05, the performance difference is significant.

## Results and Discussion

### Evaluation of Feature-Based Method by 5-fold Cross-Validation

In this section, an attempt was made to systematically evaluate the usefulness of different sequence features proposed in our study. To this end, we first established a baseline predictor that only utilized the PSSM feature as the inputs of the SVM algorithm, because this feature is most commonly utilized in the existing studies. We then gradually added the other sequence features into the baseline predictor and checked if the new predictors could achieve better performance.

Table 1 illustrates the performance of different SVM-based predictors on the DB312 and RB264 datasets. According to the residue-based evaluation, we find that the baseline predictor yielded similar performance for both DNA- and RNA-binding residues, with a F1 of around 0.49 and MCC of around 0.42. This result reveals that we can achieve acceptable performance using the PSSM alone as suggested in most previous studies. When the residue conservation scores and sequence-derived structural features were gradually integrated into the baseline predictor, the F1 and MCC measures were respectively raised by about 0.02 on both datasets, suggesting that these features contain complementary information which is not included in the PSSM. We further observe that progressive incorporation of the physicochemical properties, interface propensity, and sequential position features can marginally improve the prediction performance. Finally, when the global features were used, our feature-based prediction model (SNBRFinder^F^) reached the optimal performance with a F1 of about 0.525 and MCC of about 0.460 for both types of binding residues. Based on these results, we summarize that the residue conservation scores, predicted structural features, and global features played dominant roles in improving the performance of PSSM-based predictor, and the remaining features can also help to enhance the prediction although their contribution was not so remarkable. In comparison with the baseline predictor, additionally, the AUC values of SNBRFinder^F^ were raised from 0.835 and 0.811 to 0.862 and 0.845 on DB312 and RB264, respectively, demonstrating the superiority of our feature-based predictor according to the overall performance assessment.

**Table 1:** Residue-based evaluation of different feature-based predictors on DB312 (RB264).

Feature^a^	Recall	Precision	F1	ACC	MCC	AUC
PSSM	0.500 (0.498)	0.478 (0.492)	0.488 (0.494)	0.878 (0.865)	0.420 (0.417)	0.835 (0.811)
PSSM+CS	0.506 (0.521)	0.495 (0.498)	0.499 (0.509)	0.882 (0.866)	0.433 (0.432)	0.845 (0.822)
PSSM+CS+PS	0.515 (0.524)	0.502 (0.510)	0.507 (0.516)	0.884 (0.870)	0.442 (0.441)	0.854 (0.830)
PSSM+CS+PS+PC	0.521 (0.527)	0.502 (0.508)	0.510 (0.517)	0.884 (0.869)	0.445 (0.442)	0.856 (0.833)
PSSM+CS+PS+PC+IP	0.522 (0.528)	0.501 (0.507)	0.511 (0.517)	0.884 (0.869)	0.445 (0.441)	0.857 (0.833)
PSSM+CS+PS+PC+IP+SP	0.532 (0.529)	0.496 (0.508)	0.512 (0.518)	0.882 (0.869)	0.446 (0.443)	0.858 (0.834)
PSSM+CS+PS+PC+IP+SP+GF	0.544 (0.549)	0.505 (0.513)	0.523 (0.530)	0.885 (0.871)	0.458 (0.456)	0.862 (0.845)

^a^PSSM: position specific scoring matrix, CS: residue conservation scores, PS: predicted structural features, PC: physicochemical properties,

IP: interface propensity, SP: sequential position, and GF: global features.

Furthermore, we utilized the chain-based evaluation to estimate the predictors mentioned above. Focusing on the DB312 dataset, we see from S3 Table that the F1 and MCC measures of each predictor calculated using chain-based way were slightly higher than those generated using residue-based way, indicating that as regards DNA-binding residue prediction, all these feature-based predictors were not sensitive to different evaluation types. By contrast, as these predictors were applied to RNA-binding residues, there was a remarkable decrease in their performance according to the chain-based evaluation measures. For instance, the F1 and MCC measures of SNBRFinder^F^ drastically dropped from 0.530 and 0.456 to 0.454 and 0.343 for the RB264 dataset, respectively. Similar observations have been reported by previous studies [9, 39]. This might be due to the fact that the correctly predicted RNA-binding residues were clustered in some query sequences such as those binding to ribosomal RNAs. Even so, SNBRFinder^F^ consistently outperformed the PSSM-based predictor on both datasets. The AUC values of the former were 0.852 and 0.760 for DB312 and RB264 respectively, whereas the measures of the latter were 0.829 and 0.746. The statistical significance of performance differences is provided in S4 Table. These results confirm again the effectiveness of our additional sequence features for predicting nucleic acid-binding residues.

### Impact of Different Machine Learning Techniques and Training Sets on Feature-Based Prediction

As is well known, machine learning algorithm and training set are the major determinants of feature-based prediction. Previous studies have exploited various machine learning models, such as SVM, random forest (RF), neural networks (NN), and naive Bayes (NB), to recognize DNA- or RNA-binding residues [3, 9, 11, 13, 14, 16, 23–33, 40, 57]. On the other hand, there are two basic ways to establish the training set in the existing studies. One way is to build an unbalanced training set using all the residues [9, 23], and the other way is to establish a balanced training set by merging all the binding residues with the same number of randomly sampled nonbinding residues [8, 21, 22]. In this work, to achieve an optimal feature predictor, we combined four types of machine learning algorithms with two types of training sets to construct eight predictors and systematically compared the performance of them by conducting 5-fold cross-validation on the DB312 and RB264 datasets.

As shown in Table 2, on the residue-based level, the SVM-based predictor produced the best performance on both DB312 and RB264, followed by RF- and NN-based counterparts, whereas the NB-based predictor attained the worst performance. For example, when the above predictors were trained using the balanced positive and negative samples, the MCC measures of them were 0.418/0.426, 0.363/0.415, 0.344/0.379, and 0.257/0.277 for the DB312/RB264 dataset, respectively. It is clear that the SVM algorithm has distinct advantages over the other popular machine learning techniques. Furthermore, we observe that all these algorithms except NB achieved the relatively higher F1 and MCC measures using the unbalanced training set than using the balanced one. In particular, the SVM-based predictor yielded the F1 and MCC values of 0.523/0.530 and 0.458/0.456 (unbalanced) in comparison with 0.489/0.506 and 0.418/0.426 (balanced) on DB312/RB264. Obviously, the information loss induced by random sampling weakened the prediction performance. However, it should be pointed out that we achieved the better performance using all the training samples at the cost of taking much longer to train the prediction models. As the chain-based evaluation was considered, similar tendency can be observed in S5 Table. As a consequence, the predictor based on the combination of SVM and unbalanced training set (SNBRFinder^F^) is the optimal choice for both DNA- and RNA-binding residue predictions.

**Table 2:** Residue-based evaluation of different machine learning models and training sets on DB312 (RB264).

Training set	Classifier^a^	Recall	Precision	F1	ACC	MCC	AUC
Balanced	NB	0.625 (0.575)	0.243 (0.291)	0.349 (0.386)	0.728 (0.755)	0.257 (0.277)	0.723 (0.708)
	NN	0.482 (0.474)	0.384 (0.456)	0.426 (0.462)	0.849 (0.853)	0.344 (0.379)	0.813 (0.801)
	RF	0.487 (0.535)	0.405 (0.465)	0.442 (0.497)	0.857 (0.855)	0.363 (0.415)	0.820 (0.822)
	SVM	0.541 (0.533)	0.448 (0.481)	0.489 (0.506)	0.869 (0.862)	0.418 (0.426)	0.847 (0.832)
Unbalanced	NB	0.586 (0.590)	0.252 (0.285)	0.351 (0.383)	0.748 (0.746)	0.257 (0.274)	0.717 (0.701)
	NN	0.443 (0.489)	0.441 (0.479)	0.440 (0.482)	0.869 (0.860)	0.367 (0.403)	0.812 (0.799)
	RF	0.468 (0.519)	0.429 (0.488)	0.447 (0.502)	0.865 (0.863)	0.372 (0.424)	0.806 (0.817)
	SVM	0.544 (0.549)	0.505 (0.513)	0.523 (0.530)	0.885 (0.871)	0.458 (0.456)	0.862 (0.845)

^a^NB: naive Bayes, NN: neural networks, RF: random forest, and SVM: support vector machines.

### Evaluation of Template-Based Predictor by 5-Fold Cross-Validation

**Fig 2:**
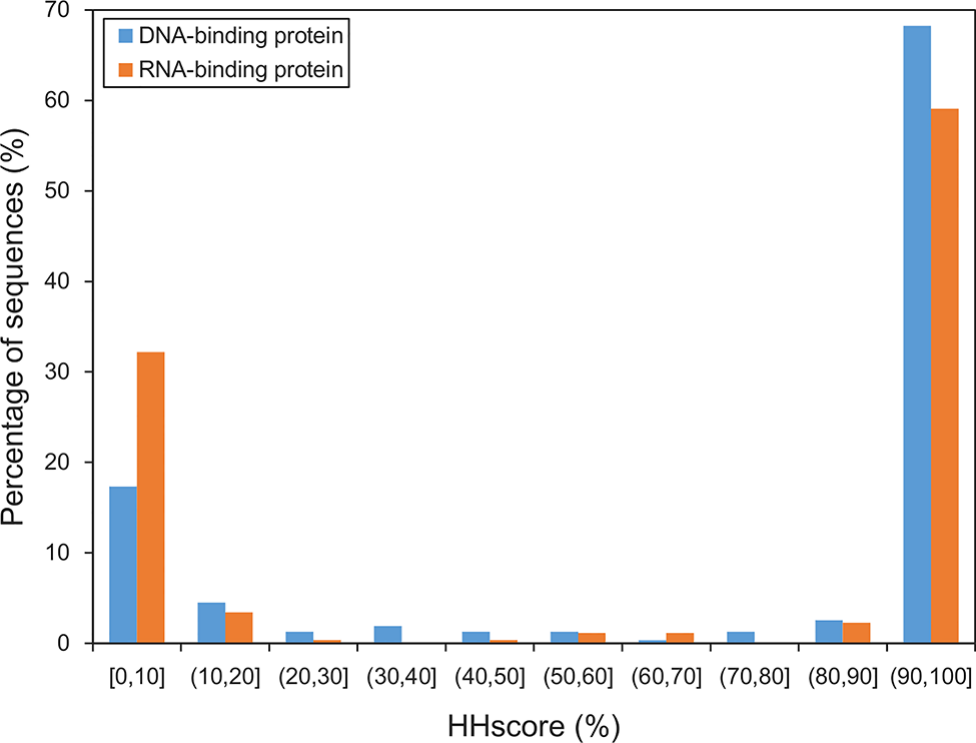
HHscore distribution of optimal templates for DB312 and RB264. The HHscore produced by HHblits ranges from 0 to 100% and is used to measure the similarity between the query sequence and its optimal template.

Additionally, the alignment-based predictor named SNBRFinder^T^ was tested on DB312 and RB264. HHscore is an important indicator to measure the degree to which the template is actually homologous to the query. Fig 2 illustrates that DNA- and RNA-binding proteins shared generally similar HHscore distribution, but the former can more readily attain a reliable template. For example, 68% of the chains in DB312 found a template with HHscore larger than 90%, whereas only 59% of the chains in RB264 can retrieve such template. When the best template was chosen, the putative binding residues can be predicted based on the alignment information between the query and template. As demonstrated in Table 3, the higher template availability of DNA-binding proteins resulted in the better performance for predicting their binding residues. Concretely, in terms of the residue-based evaluation, SNBRFinder^T^ achieved a F1 and MCC of 0.479 and 0.447 on DB312, and 0.417 and 0.378 on RB264, respectively. We also checked SNBRFinder^T^ using the chain-based evaluation. Compared to the residue-based measures, we attained the relatively lower chain-based measures on DB312, with a F1 and MCC of 0.435 and 0.397. Unlike our feature-based prediction, the template-based method was sensitive to different evaluation types for DNA-binding proteins. As for RB264, SNBRFinder^T^ yielded the remarkably worse F1 and MCC values (0.305 and 0.240), which was similar to the tendency of SNBRFinder^F^. In summary, the HMM-based search can be effectively utilized to capture the remote template of query sequence, which provides another approach to annotate nucleic acid-binding residues only using sequence information.

**Table 3:** Evaluation of our template-based predictor on DB312 (RB264).

Evaluation	Recall	Precision	F1	ACC	MCC
Residue-based	0.382 (0.321)	0.642 (0.594)	0.479 (0.417)	0.903 (0.880)	0.447 (0.378)
Chain-based	0.412 (0.287)	0.522 (0.379)	0.435 (0.305)	0.881 (0.817)	0.397 (0.240)

### Evaluation of Our Hybrid Predictor by 5-Fold Cross-Validation

Once the individual prediction models were established, we can use a piecewise function to merge the outputs of them as suggested in Materials and Method section. Here we compared the performance of our individual and hybrid approaches on DB312 and RB264. As shown in Table 4, when the HHscore cutoff was assigned to 85%, we can retrieve 216 DNA-binding proteins (DB216) and 159 RNA-binding proteins (RB159), respectively. It is clear that our template predictor attained good performance on both subsets, because most of these sequences can find really homologous template for inferring their putative binding residues. The MCC values of SNBRFinder^T^ on DB216 and RB159 were 0.539 and 0.493, respectively. Meanwhile, our feature predictor SNBRFinder^F^ produced slightly lower performance, yielding the MCC values of 0.506 and 0.471 for these two subsets. Accordingly, we propose that these sequences belonged to the easy class, in which their binding residues, at least in part, can correctly recognized by both template and feature methods. Compared to the component predictors, the MCC values of SNBRFinder was improved to 0.563 and 0.544, suggesting that the prediction results of SNBRFinder^T^ and SNBRFinder^F^ contained complementary binding signatures.

**Table 4:** Residue-based evaluation of our individual and hybrid predictors on DB312 (RB264).

Group	NO. of chains	Method	Recall	Precision	F1	ACC	MCC	AUC
HHscore ≥ 85%	216 (159)	SNBRFinder^F^	0.596 (0.548)	0.539 (0.530)	0.565 (0.537)	0.894 (0.882)	0.506 (0.471)	0.883 (0.849)
		SNBRFinder^T^	0.513 (0.474)	0.666 (0.635)	0.580 (0.541)	0.915 (0.899)	0.539 (0.493)	N/A (N/A)
		SNBRFinder	0.641 (0.606)	0.591 (0.598)	0.614 (0.601)	0.907 (0.900)	0.563 (0.544)	0.900 (0.875)
HHscore < 85%	96 (105)	SNBRFinder^F^	0.422 (0.550)	0.421 (0.485)	0.420 (0.514)	0.861 (0.843)	0.342 (0.423)	0.806 (0.830)
		SNBRFinder^T^	0.074 (0.042)	0.383 (0.262)	0.123 (0.073)	0.877 (0.837)	0.125 (0.049)	N/A (N/A)
		SNBRFinder	0.437 (0.550)	0.416 (0.485)	0.424 (0.514)	0.859 (0.843)	0.345 (0.423)	0.806 (0.830)
All	312 (264)	SNBRFinder^F^	0.544 (0.549)	0.505 (0.513)	0.523 (0.530)	0.885 (0.871)	0.458 (0.456)	0.862 (0.845)
		SNBRFinder^T^	0.382 (0.321)	0.642 (0.594)	0.479 (0.417)	0.903 (0.880)	0.447 (0.378)	N/A (N/A)
		SNBRFinder	0.581 (0.586)	0.538 (0.555)	0.558 (0.570)	0.893 (0.883)	0.498 (0.502)	0.877 (0.863)

As for the remaining 96 DNA-binding proteins (DB96) and 105 RNA-binding proteins (RB105), the MCC values of SNBRFinder^T^ were 0.125 and 0.049, respectively. Obviously, the template unavailability for most sequences in these subsets resulted in the poor prediction of their binding residues. Focusing on the feature-based prediction, we find that the performance on DB96/RB105 was clearly inferior to that on DB216/RB159, suggesting that the binding residues in these query sequences whose remote template cannot be found by HMM-based search may be also more difficultly identified by feature-based method. Even so, SNBRFinder^F^ output an acceptable MCC value of 0.342 for DB96 and 0.423 for RB105, respectively. Due to the fact that the performance of SNBRFinder^T^ on these two subsets was close to a random guess, the combined predictor SNBRFinder just relied on the outputs of SNBRFinder^F^. With regard to the whole datasets, SNBRFinder also outperformed SNBRFinder^F^ and SNBRFinder^T^, yielding a MCC value of 0.498/0.502 against 0.458/0.456 and 0.447/0.378 for DB312/RB264. Revisiting Table 1, we can see that, compared to the PSSM-based predictor, the MCC value of SNBRFinder for DNA- and RNA-binding residue prediction was increased by 0.072 and 0.085, respectively, showing the distinguished advantage of our hybrid algorithm over the baseline predictor. Similar tendency can be seen for chain-based evaluation measures in S6 Table. Notably, there still exists a distinct performance gap as SNBRFinder was estimated using different evaluation types on RNA-binding proteins, but this phenomenon did not appear for DNA-binding proteins. The above results suggest that the individual prediction model can indeed provide additional information between each other and the complementary relationship between them is helpful for improving sequence-based nucleic acid-binding residue prediction.

### Evaluation of Proposed Prediction Models Using Various Datasets

To further check the effectiveness and robustness of our method, we tested our individual and integrative predictors using a group of datasets prepared by previous studies (e.g., DB123, DB232, DB374, RB106, RB144, and RB198). The PSSM-based predictor was still considered as the baseline. Note that the binding residues of each dataset were defined using the original criterion in the reference. Detailed results about different datasets are shown in Table 5. As regards the baseline predictor, we find that there exists obvious performance discrepancy between the three DNA-binding protein datasets, while the three RNA-binding protein datasets collected by the same research group shared largely similar performance. For all these datasets, however, as our additional sequence features were incorporated into the baseline predictor, the MCC value of the feature predictor SNBRFinder^F^ can be raised by 0.03~0.06, confirming again the usefulness of these features. Focusing on the template-based prediction, we can see that the performances on these datasets were not as well as those on DB312 and RB264, which is probably due to the relatively small size of the template library. Despite this limitation, the MCC values of these datasets ranged from 0.258 to 0.415, further suggesting that the sequence-based template approach can be utilized to predict both DNA- and RNA-binding residues. Furthermore, when the individual predictors were integrated, SNBRFinder achieved superior performance for each dataset. In particular, the AUC measures of SNBRFinder were around 0.045 higher than those of the PSSM-based predictor. In addition, as illustrated in S7 Table, the above tendency can be observed using chain-based evaluation as well as using residue-based evaluation. Altogether, the comprehensive validation indicates that our proposed algorithm can be effectively applied to different datasets.

**Table 5:** Residue-based evaluation of our proposed predictors on various datasets.

Dataset	Method	Recall	Precision	F1	ACC	MCC	AUC
DB123	PSSM	0.481	0.374	0.415	0.836	0.330	0.799
	SNBRFinder^F^	0.532	0.416	0.465	0.853	0.386	0.831
	SNBRFinder^T^	0.223	0.587	0.322	0.886	0.313	N/A
	SNBRFinder	0.547	0.436	0.482	0.859	0.407	0.839
DB232	PSSM	0.499	0.288	0.365	0.871	0.313	0.812
	SNBRFinder^F^	0.520	0.336	0.407	0.887	0.359	0.846
	SNBRFinder^T^	0.251	0.477	0.329	0.924	0.310	N/A
	SNBRFinder	0.544	0.353	0.428	0.892	0.382	0.855
DB374	PSSM	0.418	0.499	0.453	0.931	0.419	0.846
	SNBRFinder^F^	0.507	0.492	0.499	0.930	0.461	0.881
	SNBRFinder^T^	0.373	0.541	0.440	0.935	0.415	N/A
	SNBRFinder	0.517	0.538	0.526	0.936	0.492	0.889
RB106	PSSM	0.540	0.518	0.527	0.819	0.416	0.803
	SNBRFinder^F^	0.574	0.531	0.551	0.824	0.442	0.823
	SNBRFinder^T^	0.179	0.602	0.272	0.825	0.258	N/A
	SNBRFinder	0.630	0.571	0.599	0.841	0.501	0.852
RB144	PSSM	0.529	0.511	0.518	0.820	0.409	0.801
	SNBRFinder^F^	0.578	0.533	0.554	0.830	0.450	0.829
	SNBRFinder^T^	0.261	0.605	0.363	0.834	0.319	N/A
	SNBRFinder	0.644	0.553	0.594	0.839	0.497	0.849
RB198	PSSM	0.491	0.475	0.482	0.844	0.391	0.795
	SNBRFinder^F^	0.517	0.501	0.509	0.852	0.422	0.825
	SNBRFinder^T^	0.274	0.618	0.380	0.867	0.350	N/A
	SNBRFinder	0.566	0.547	0.556	0.866	0.477	0.848

### Comparison with Other Sequence- and Structure-Based Predictors

In addition to the HMM-based predictor, we established several other template predictors using the existing sequence or structure alignment programs, such as BLAST [35], TMalign [58], and SPalign [18]. Once we acquired the optimal template of the query protein using these alignment tools, the putative binding residues can be determined in terms of the sequence or structure alignment between the query and template. These predictors were also evaluated using DB312 and RB264. As shown in Table 6, the F1 and MCC values of the BLAST-based predictor were around 0.32 and 0.31 for both datasets based on the residue-based evaluation, which were clearly worse than those of our HMM-based method (Table 4). The major reason might be that the profile alignment (HHblits) can more effectively retrieve the remote template than the simple sequence alignment (BLAST). With respect to the structure-based predictors, TMalign/SPalign attained the F1 and MCC values of 0.474/0.488 and 0.434/0.447 on the DB312 dataset, and 0.366/0.432 and 0.317/0.381 on the RB264 dataset, respectively. It is clear that HHblits was comparable to SPalign and superior to TMalign. On the chain-based level (S6 and S8 Tables), HHblits achieved similar performance to TMalign, and was slightly worse than SPalign. These suggest that we could replace structure alignment with HMM alignment for template-based binding residue prediction.

**Table 6:** Residue-based evaluation of other sequence- and structure-based predictors on DB312 (RB264).

Type	Method^a^	Recall	Precision	F1	ACC	MCC	AUC
Template	BLAST	0.222 (0.218)	0.577 (0.613)	0.321 (0.322)	0.891 (0.878)	0.311 (0.315)	N/A (N/A)
	TMalign	0.389 (0.284)	0.606 (0.516)	0.474 (0.366)	0.900 (0.869)	0.434 (0.317)	N/A (N/A)
	SPalign	0.407 (0.350)	0.611 (0.566)	0.488 (0.432)	0.901 (0.878)	0.447 (0.381)	N/A (N/A)
Feature	NBRFeature^SEQ^	0.588 (0.601)	0.525 (0.515)	0.555 (0.554)	0.890 (0.871)	0.493 (0.482)	0.879 (0.865)
	NBRFeature^STR^	0.595 (0.556)	0.435 (0.501)	0.502 (0.526)	0.863 (0.867)	0.432 (0.450)	0.858 (0.852)
	NBRFeature	0.607 (0.594)	0.525 (0.539)	0.563 (0.564)	0.890 (0.878)	0.502 (0.495)	0.888 (0.872)
Hybrid	SNBRFinder^BLAST^	0.539 (0.554)	0.520 (0.541)	0.528 (0.547)	0.888 (0.878)	0.466 (0.477)	0.867 (0.854)
	NBRDetector^TMalign^	0.650 (0.606)	0.542 (0.580)	0.591 (0.592)	0.895 (0.889)	0.534 (0.528)	0.900 (0.886)
	NBRDetector	0.654 (0.615)	0.545 (0.586)	0.594 (0.599)	0.896 (0.891)	0.538 (0.537)	0.902 (0.889)

^a^NBRFeature: a structure-based feature predictor in our previous work, NBRDetector: the combination of NBRFeature and SPalign, NBRDetector^TMalign^:

the combination of NBRFeature and TMalign, and SNBRFinder^BLAST^: the combination of SNBRFinder^F^ and BLAST.

We next applied the structure-based feature predictors proposed in our previous study to the current datasets. The evaluated predictors contain NBRFeature^SEQ^, NBRFeature^STR^, and NBRFeature, where SEQ and STR represent the sequential and structural microenvironment respectively, and NBRFeature is a hybrid feature predictor. More details can be found in our earlier publication [22]. As given in Tables 4 and 6, for both DB312 and RB264, the performance of our sequence-based predictor SNBRFinder^F^ was slightly better than that of NBRFeature^STR^, but was relatively worse than that of NBRFeature^SEQ^. From these results, we can propose that the sequential window was more effective for characterizing the neighborhood of a target residue compared to the structural window, and that the performance of NBRFeature^SEQ^ might be the upper limit of nucleic acid-binding residue prediction based on sequence features. Additionally, it can be observed that the MCC value of SNBRFinder^F^ (Table 4) was around 0.04 lower than that of NBRFeature for both datasets. Even though our sequence-based feature predictor was relatively inferior to the structure-based counterpart, it could be considered as an alternative solution if the structural characteristics of query protein are not available.

We further constructed several hybrid prediction models based on the aforementioned predictors. As SNBRFinder^F^ and BLAST were merged, the performance of this sequence-based hybrid predictor was slightly improved compared to that of SNBRFinder^F^, yielding a F1 and MCC of 0.528 and 0.466 for DB312, and 0.547 and 0.477 for RB264, respectively. All these measures were around 0.03 lower than the corresponding measures of SNBRFinder (Table 4). Despite the fact that we achieved an acceptable performance using BLAST alone, the outputs of BLAST were just marginally complementary to those of SNBRFinder^F^. As a component predictor, HHblits also showed its advantage over BLAST. Focusing on the structure-based prediction, we observe that the NBRDetector algorithm (the combination of NBRFeature and SPalign) outperformed SNBRFinder by around 0.035 in terms of the F1 and MCC measures for both datasets. The chain-based evaluation measures for these predictors are provided in S8 Table, and the statistical significance of performance differences is given in S9 Table. Because NBRDetector is one of the best structure-based algorithms and the performance discrepancy between SNBRFinder and NBRDetector was relatively small, we believe that our sequence-based hybrid strategy paves a novel way for accurately predicting DNA- and RNA-binding residues.

### Comparison with Structural Model-Based Prediction

Given a query sequence, another way for annotating its nucleic acid-binding residues is to leverage the information from its structural model. To this end, the predicted structures of 35 DNA-binding proteins (DB35) and 36 RNA-binding proteins (RB36) prepared by Dror et al. [41] were used as independent datasets to test our previous structure-based predictors, the results of which were to be compared with those of our sequence-based predictors. From Table 7, it can be seen that our sequence-based hybrid algorithm consistently outperformed its component predictors for both DNA- and RNA-binding proteins. For example, the MCC values of SNBRFinder, SNBRFinder^F^, and SNBRFinder^T^ were 0.491, 0.457, and 0.375 for DB35, and 0.498, 0.461, and 0.290 for RB36, respectively. When the native structures were used, as expected, the structure-based individual and hybrid predictors (except the template predictor tested on DB35) were generally superior to their sequence-based counterparts. For instance, the MCC values of NBRDetector, NBRFeature, and NBRTemplate were 0.530, 0.500, and 0.370 for DB35, and 0.516, 0.480, and 0.330 for RB36, respectively. These results further indicate that our sequence-based predictors can achieve competitive performance in comparison with the state-of-the-art algorithms depending on structural information. If the native structures were replaced with the simulated structures generated by the I-TASSER software, the performance of structure-based prediction for DNA-binding residues degraded remarkably, whereas the performance for RNA-binding residues just slightly decreased. In this case, the MCC values of NBRDetector, NBRFeature, and NBRTemplate were 0.476, 0.451, and 0.342 for DB35, and 0.501, 0.463, and 0.307 for RB36, respectively. It is clear that our sequence-based prediction yielded better performance than the prediction based on structural models for DNA-binding proteins, and attained comparable performance for RNA-binding proteins. Similar observation can be found for the chain-based evaluation in S10 Table. The statistical tests further confirm the aforementioned results (S11 Table). Additionally, a distinct disadvantage of the structural model-based prediction is that it might spend too much time on the structure prediction for query sequence, but our proposed sequence-based approach dose not suffer this problem. Thus, the SNBRFinder algorithm is an effective and efficient tool for finding nucleic acid-binding residues in query sequence.

**Table 7:** Residue-based evaluation of sequence- and structural model-based predictions on DB39 (RB36).

Type^a^	Method	Recall	Precision	F1	ACC	MCC	AUC
Structure	NBRFeature	0.606 (0.659)	0.543 (0.538)	0.573 (0.593)	0.874 (0.817)	0.500 (0.480)	0.887 (0.848)
	NBRTemplate^b^	0.326 (0.297)	0.578 (0.599)	0.417 (0.397)	0.873 (0.818)	0.370 (0.330)	N/A (N/A)
	NBRDetector	0.639 (0.665)	0.563 (0.579)	0.598 (0.619)	0.881 (0.835)	0.530 (0.516)	0.893 (0.865)
Model	NBRFeature	0.553 (0.673)	0.509 (0.510)	0.530 (0.581)	0.864 (0.804)	0.451 (0.463)	0.851 (0.843)
	NBRTemplate	0.300 (0.292)	0.553 (0.561)	0.389 (0.384)	0.869 (0.812)	0.342 (0.307)	N/A (N/A)
	NBRDetector	0.582 (0.684)	0.524 (0.548)	0.552 (0.609)	0.869 (0.823)	0.476 (0.501)	0.860 (0.857)
Sequence	SNBRFinder^F^	0.568 (0.644)	0.508 (0.523)	0.536 (0.577)	0.864 (0.810)	0.457 (0.461)	0.844 (0.832)
	SNBRFinder^T^	0.312 (0.279)	0.607 (0.545)	0.412 (0.369)	0.876 (0.808)	0.375 (0.290)	N/A (N/A)
	SNBRFinder	0.591 (0.666)	0.539 (0.556)	0.564 (0.606)	0.873 (0.825)	0.491 (0.498)	0.861 (0.854)

^a^Structure: native structure, Model: structural model, and Sequence: protein sequence.

^b^NBRTemplate: the template predictor implemented with SPalign.

## Comparison with Existing Sequence-Based Methods

In this section, we compared our proposed predictors with the existing sequence-based approaches for DNA- and/or RNA-binding residue prediction. Three datasets collected in the recent review articles (e.g., DB33, RB49, and RB44) were respectively used as independent dataset to compare different algorithms. For each dataset, we utilized the evaluation measures proposed in the original work to estimate our method. As a result, the performance on DB33 and RB49 was evaluated using the chain-based way, whereas the performance on RB44 was assessed using the residue-based way.

**Fig 3:**
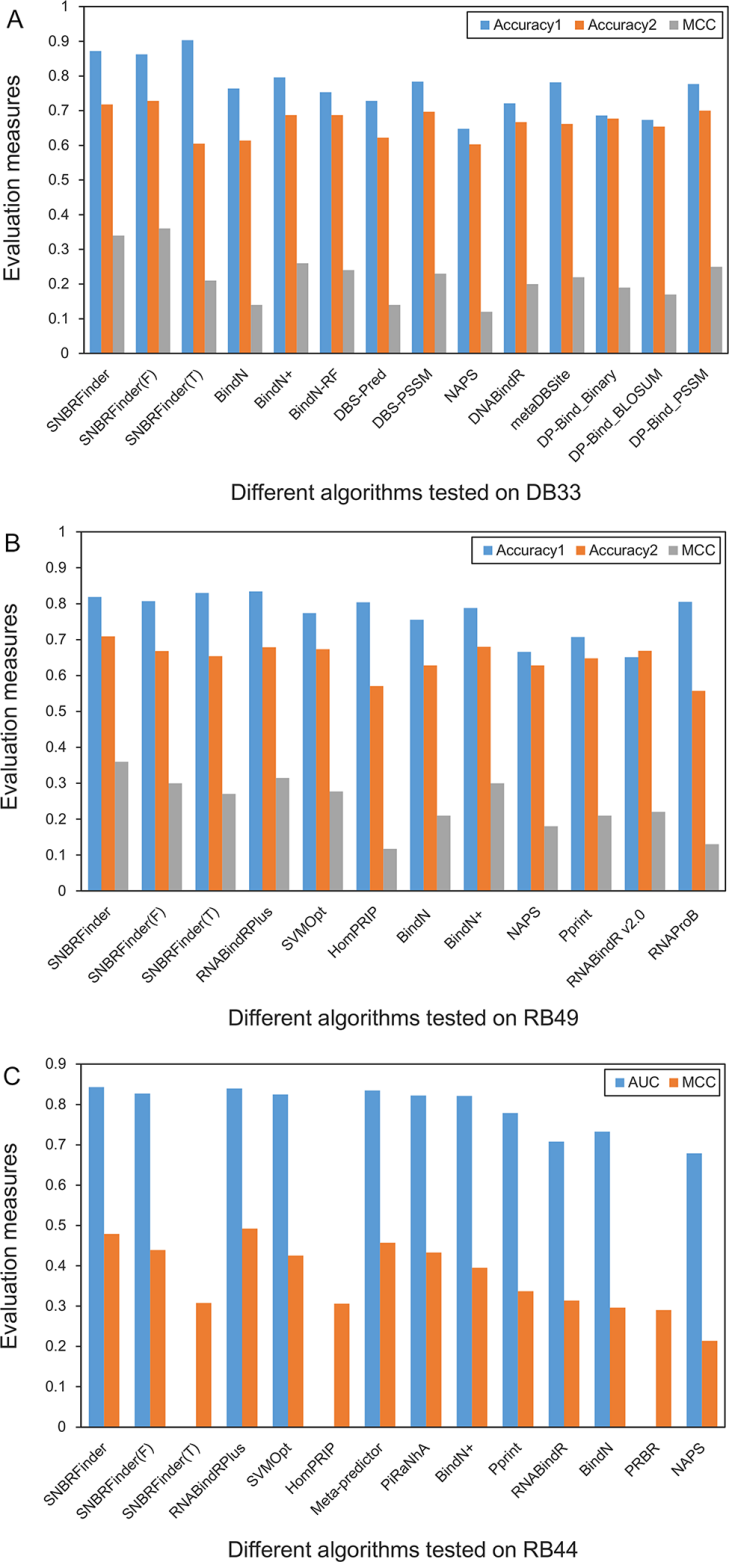
Comparison of our algorithms and existing approaches on three datasets. (A) DB33, (B) RB49, (C) RB44. In (A) and (B), Accuracy1 = (TP+TN)/(TP+TN+FP+FN) and Accuracy2 = (Sensitivity+Specificity)/2. In (C), the AUC values of SNBRFinder^T^, HomPRIP, and PRBR are not provided, because the outputs of these three predictors are binary values. With the exception of SNBRFinder and RNABindRPlus (including the component predictors), the evaluation measures of the other approaches are derived from the recent review articles.

From Fig 3A, we find that the combination of our template- and feature-based outputs resulted in the degraded performance as our predictors were tested on DB33. The MCC values of SNBRFinder^T^, SNBRFinder^F^, and SNBRFinder were 0.21, 0.36, and 0.34, respectively. The reason might be that the HMM-based prediction on this dataset was relatively worse and thus the prediction results were not complementary to those of our feature-based predictor. Even so, our hybrid predictor showed distinct superiority over the existing approaches. For instance, the MCC value of SNBRFinder was about 0.06 higher than that of BindN+ and BindN-RF, which achieved the best performance among the competing algorithms. Furthermore, SNBRFinder^F^ and SNBRFinder yielded the higher Accuracy1 and Accuracy2 measures. The results suggest that the combination of our sequence features is more effective for detecting DNA-binding residues compared to the feature sets used in the existing studies.

Fig 3B shows the performance of different RNA-binding residue predictors on RB49. Our hybrid strategy adequately showed its effectiveness on this dataset. The MCC values of SNBRFinder^T^, SNBRFinder^F^, and SNBRFinder were 0.27, 0.30, and 0.36, respectively. Similar to SNBRFinder, RNABindRPlus is also composed of a feature-based predictor (SVMOpt) and a template-based predictor (HomPRIP). To make a fair comparison, the 30% sequence identity threshold was used to discard the close homologs in HomPRIP. The MCC values of HomPRIP, SVMOpt, and RNABindRPlus were 0.12, 0.28, and 0.32, respectively. It is evident that our template predictor remarkably outperformed the corresponding counterpart of RNABindRPlus. This is probably due to the fact that HomPRIP chose BLAST as the search engine which could not sensitively recognize the remote templates for most queries in RB49. In comparison, the HMM-based search performed relatively well on this dataset. Our feature-based predictor also showed slightly better performance than SVMOpt which only uses PSSM as the inputs. This might be due to the usefulness of our additional sequence features. As expected, the superiority of our component predictors resulted in the better performance of SNBRFinder. In Fig 3B, we also observe that the Accuracy1, Accuracy2, and MCC measures of SNBRFinder were superior to those of other feature-based algorithms (e.g., BindN+ and RNABindR v2.0), further showing the advantage of our method over the current state-of-the-arts. It should be pointed out that the performance of existing methods on DB33 and RB49 could be overestimated, because Nagarajan et al. [43, 44] did not use a stringent sequence identity cutoff such as 30% to remove the redundancy between the test set and the training sets (private communications).

The RB44 dataset prepared by Puton et al. [45] was further used to check our method. The comparison with other predictors on this dataset is shown in Fig 3C. The MCC values of SNBRFinder^T^, SNBRFinder^F^, and SNBRFinder were 0.308, 0.439, and 0.479, respectively, while the corresponding measures of HomPRIP, SVMOpt, and RNABindRPlus were 0.306, 0.425, and 0.492, respectively. According to the residue-based evaluation, RNABindRPlus was slightly better than SNBRFinder. When the chain-based way was used, however, SNBRFinder achieved relatively higher performance compared to RNABindRPlus (S12 Table). The statistical significance of performance differences between them is provided in S13 Table. On the other hand, in terms of the MCC measure, our hybrid predictor SNBRFinder not only beat the existing feature-based predictors (e.g., PiRaNhA, BindN+, and PPRInt), but also outperformed the meta-predictor composed of them. Furthermore, SNBRFinder generated the highest AUC value (0.843) among all the evaluated predictors. The better performance attained by our approach mainly resulted from two aspects: (i) the HMM-based search was specifically utilized to find the weakly homologous template of nucleic acid-binding sequences, which laid a solid foundation for our sequence-based template and hybrid algorithms; (ii) the comprehensive feature descriptors proposed in our study can reasonably capture the sequence pattern of the binding residues, which provided another solution for the query sequences whose remote templates are not available.

### Evaluation of Our Method with Non-Nucleic Acid Binding Sequences

**Fig 4:**
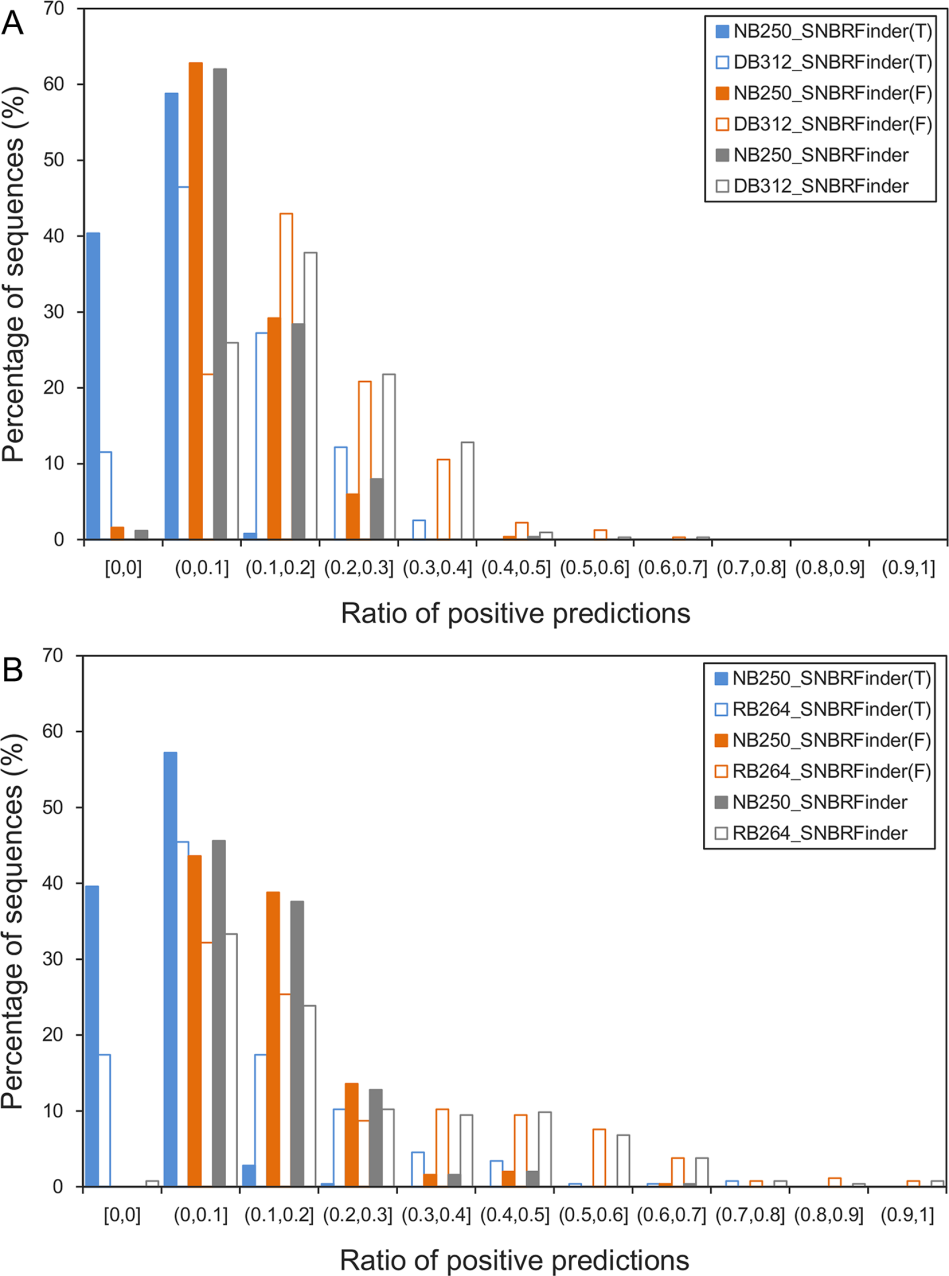
Distribution of the ratio of positive predictions for non-nucleic acid binding and nucleic acid binding proteins. (A) NB250 annotated by our predictors trained with DB312, (B) NB250 annotated by our predictors trained with RB264. The solid bars represent the prediction results of non-nucleic acid binding sequences, while the hollow bars represent the prediction results of nucleic acid binding sequences.

Finally, the non-nucleic acid binding protein dataset (NB250) prepared by Stawiski et al. [59] was used to check our predictors, which were trained with DB312/RB264. The results of 5-fold cross-validation on DB312/RB264 were considered as a control. For each sequence, we calculated the ratio of positive predictions (RPP), which is defined as the number of the predicted binding residues divided by the number of all the residues. As shown in Fig 4, for all the three predictors, the distribution of the RPP of NB250 was significantly different from that of DB312/RB264 (*P-*value≤2.87×10^−10^, Kolmogorov-Smirnov test). Generally, the nonbinding sequences achieved the relatively lower RPP values compared to the real binding sequences. In particular, when SNBRFinder and SNBRFinder^F^ were applied to the RB264 dataset, around 20% of RNA-binding proteins, most of which actually bind to ribosomal RNAs, attained a RPP value greater than 0.4. Nevertheless, very few nonbinding proteins in NB250 can achieve such a high value, which reasonably showed the specificity of our proposed methods. Additionally, it should be emphasized that the majority of non-nucleic acid binding proteins yielded a RPP value greater than 0, suggesting that our methods tend to incorrectly recognize some nucleic acid-binding residues in these sequences. Hence, as the new sequences with unknown function are to be annotated by our predictors, we should carefully check the prediction results.

### SNBRFinder As a Web Service

**Fig 5:**
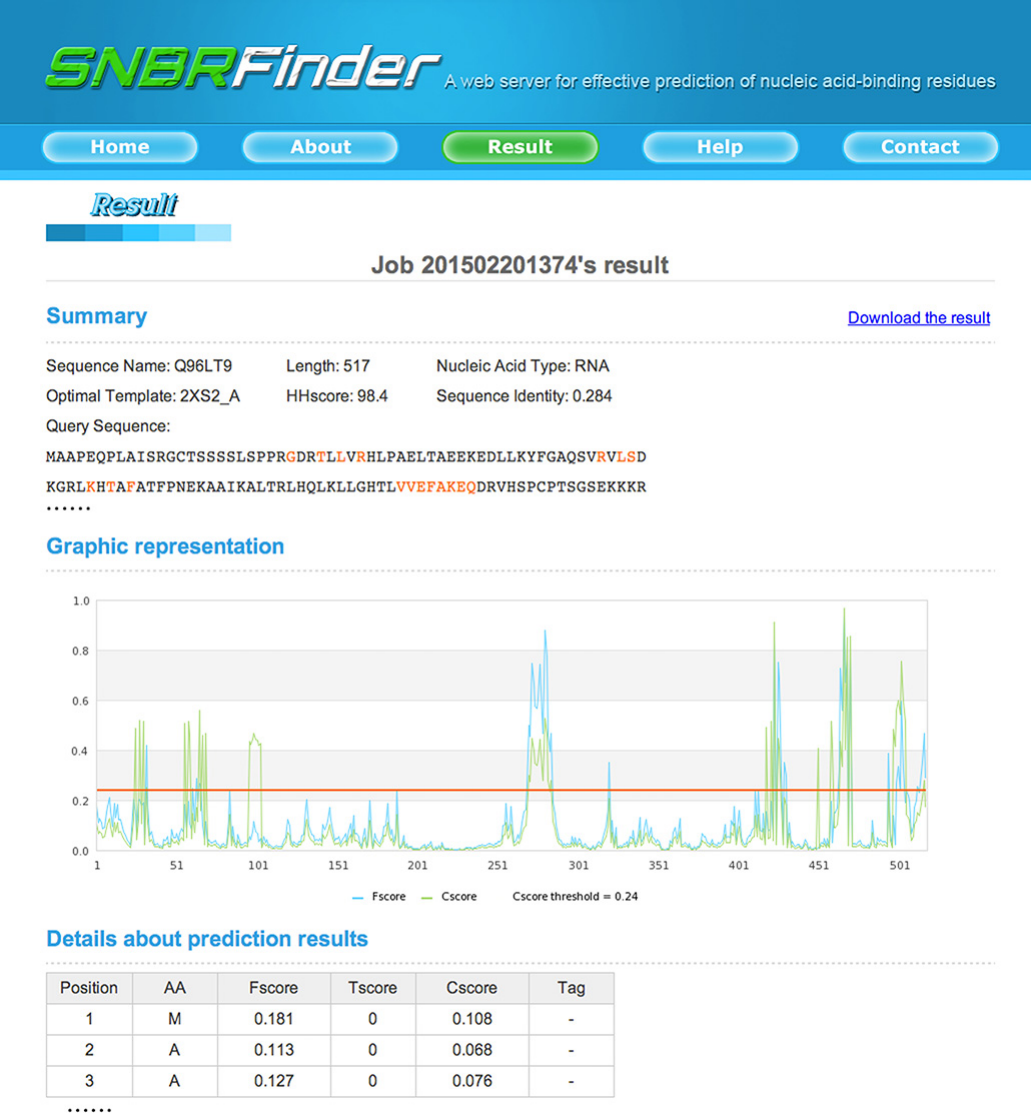
Snapshots of SNBRFinder web server. The submission page allows users to input mutiple protein sequences and specify the binding nucleic acid type. When the submitted job is finished, SNBRFinder will demonstrate the prediction results from three perspectives. The first section provides summary information about the query sequence and its optimal template. The second section is graphical representation of the prediction results. The last section includes details about the prediction results such as the outputs from our three predictors.

To highlight the value of our algorithm, we implemented SNBRFinder as a user-friendly web server freely available at http://ibi.hzau.edu.cn/SNBRFinder, which was developed in PHP, Perl-CGI, and JpGraph. Our server allows users to conveniently submit multiple queries each time with three diverse approaches including: (i) paste amino acid sequences in FASTA format; (ii) upload a sequence file from the local machine; (iii) input UniProt IDs for retrieving the remote sequence file. Meanwhile, users can choose the binding nucleic acid type they may be interested in. As the submitted job is done, SNBRFinder will demonstrate the prediction results from three perspectives (Fig 5). The first section provides summary information about the query sequence and its optimal template. The second section is graphical representation of the prediction results. The last section includes details about the prediction results from our individual and integrative predictors.

## Conclusions

Inspired by our earlier findings that both DNA- and RNA-binding residue predictions can be remarkably improved by merging structure-based template and feature methods, here we developed a sequence-based hybrid algorithm SNBRFinder for predicting nucleic acid-binding residues. To address this problem, the profile HMM alignment was ingeniously utilized to establish the sequence-based template method, which can effectively recognize the weakly homologous template of query protein as the structure-based template methods did. We also characterized each residue with comprehensive sequence features to establish an enhanced sequence-based feature predictor, which showed clear superiority over the simple PSSM-based method. Based on the complementary nature between the individual predictors, SNBRFinder yielded an obvious improvement in the performance of nucleic acid-binding residue prediction. Especially, our sequence-based prediction can achieve competitive performance in comparison with the prediction relying on the native and predicted structures. Furthermore, the comparison with current state-of-the-art algorithms demonstrates that SNBRFinder not only has advantages over the similar method such as RNABindRplus, but also remarkably outperformed other existing feature-based predictors. This work strongly suggests that our proposed methodology might open a new avenue to annotate other functional residues from protein sequence.

## Supporting Information

S1 Table Summary of thirteen datasets used in this study. (DOC)

S2 Table Physicochemical properties of different amino acids. (DOC)

S3 Table Chain-based evaluation of different feature-based predictors on DB312 (RB264). (DOC)

S4 Table Statistical significance of performance differences between different predictors developed in this study. (XLSX)

S5 Table Chain-based evaluation of different machine learning models and training sets on DB312 (RB264). (DOC)

S6 Table Chain-based evaluation of our individual and hybrid predictors on DB312 (RB264). (DOC)

S7 Table Chain-based evaluation of our proposed predictors on various datasets. (DOC)

S8 Table Chain-based evaluation of other sequence- and structure-based predictors on DB312 (RB264). (DOC)

S9 Table Statistical significance of performance differences between our sequence-based methods and the other sequence- and structure-based counterparts. (XLSX)

S10 Table Chain-based evaluation of sequence- and structural model-based predictions on DB35 (RB36). (DOC)

S11 Table Statistical significance of performance differences between sequence- and structural model-based predictions. (XLSX)

S12 Table Comparison of SNBFinder and RNABindRPlus on RB44. (DOC)

S13 Table Statistical significance of performance differences between SNBRFinder and RNABindRPlus. (XLSX)
