# Supplementary material for: SNBRFinder: A Sequence-Based Hybrid Algorithm for Enhanced Prediction of Nucleic Acid-Binding Residues
Source: PLoS One. 2015 Jul 15;10(7):e0133260. doi: 10.1371/journal.pone.0133260 (PMC4503397; doi:10.1371/journal.pone.0133260)
Supplement: S1 Table — (DOC) [file pone.0133260.s001.doc]

**S1 Table.** Summary of thirteen datasets used in this study

| Dataset | NO. of chains | Pairwise sequence identity | Distance cutoff | Binding residues | Nonbinding residues | Ratio^a^ | Reference^b^ |
| --- | --- | --- | --- | --- | --- | --- | --- |
| DB312 | 312 | 30% | 4.5 Å | 8596 | 65162 | 11.70% | Liu and Hu, 2013 |
| RB264 | 264 | 30% | 4.5 Å | 8995 | 58541 | 13.30% | Yang et al., 2014 |
| DB123 | 123 | 25% | 4.5 Å | 3152 | 23389 | 11.90% | Liu and Hu, 2013 |
| DB232 | 232 | 30% | 3.5 Å | 4168 | 52042 | 7.40% | Si et al., 2011 |
| DB374 | 374 | 25% | 3.5 Å | 5549 | 74997 | 6.90% | Wu et al., 2009 |
| RB106 | 106 | 30% | 5.0 Å | 4534 | 20172 | 18.40% | Walia et al., 2012 |
| RB144 | 144 | 30% | 5.0 Å | 6107 | 27511 | 18.20% | Walia et al., 2012 |
| RB198 | 198 | 30% | 5.0 Å | 7950 | 45710 | 14.80% | Walia et al., 2012 |
| DB35 | 35 | 25% | 4.5 Å | 837 | 5189 | 13.90% | Dror et al., 2012 |
| RB36 | 36 | 25% | 4.5 Å | 1184 | 4691 | 20.20% | Dror et al., 2012 |
| DB33 | 33 | 30% | 3.5 Å | 553 | 9517 | 5.50% | Nagarajan et al., 2013 |
| RB49 | 49 | 30% | 3.5 Å | 1223 | 11122 | 9.90% | Nagarajan and Gromiha, 2014 |
| RB44 | 44 | 40% | 3.5 Å | 1284 | 5193 | 19.80% | Puton et al., 2012 |

^a^Ratio denotes the percentage of binding residues in all the samples.

^b^The references can be found in our manuscript.
