# Supplementary material for: SNBRFinder: A Sequence-Based Hybrid Algorithm for Enhanced Prediction of Nucleic Acid-Binding Residues
Source: PLoS One. 2015 Jul 15;10(7):e0133260. doi: 10.1371/journal.pone.0133260 (PMC4503397; doi:10.1371/journal.pone.0133260)
Supplement: S2 Table — (DOC) [file pone.0133260.s002.doc]

**S2 Table. Physicochemical properties of different amino acids**

| Amino acid | Hydrophobicity | Hydrophilicity | Number of electrostatic charge | Number of potential hydrogen bonds | Isoelectric points |
| --- | --- | --- | --- | --- | --- |
| A | 0.25 | 3 | 0 | 2 | 6.11 |
| C | 0.04 | -1 | 0 | 2 | 6.31 |
| D | -0.72 | 3 | -1 | 4 | 5.945 |
| E | -0.62 | 3 | -1 | 4 | 5.785 |
| F | 0.61 | -2.5 | 0 | 2 | 5.755 |
| G | 0.16 | 0 | 0 | 2 | 6.065 |
| H | -0.4 | -0.5 | 0 | 4 | 5.565 |
| I | 0.73 | -1.8 | 0 | 2 | 6.04 |
| K | -1.1 | 3 | 1 | 2 | 5.61 |
| L | 0.53 | -1.8 | 0 | 2 | 6.035 |
| M | 0.26 | -1.3 | 0 | 2 | 5.705 |
| N | -0.64 | 0.2 | 0 | 4 | 5.43 |
| P | -0.07 | 0 | 0 | 2 | 6.295 |
| Q | -0.69 | 0.2 | 0 | 4 | 5.65 |
| R | -1.76 | -0.5 | 1 | 4 | 5.405 |
| S | -0.26 | 0.3 | 0 | 4 | 5.7 |
| T | -0.18 | -0.4 | 0 | 4 | 5.595 |
| V | 0.54 | -1.5 | 0 | 2 | 6.015 |
| W | 0.37 | -3.4 | 0 | 3 | 5.935 |
| Y | 0.02 | -2.3 | 0 | 3 | 5.705 |
