# Supplementary material for: SNBRFinder: A Sequence-Based Hybrid Algorithm for Enhanced Prediction of Nucleic Acid-Binding Residues
Source: PLoS One. 2015 Jul 15;10(7):e0133260. doi: 10.1371/journal.pone.0133260 (PMC4503397; doi:10.1371/journal.pone.0133260)
Supplement: S3 Table — (DOC) [file pone.0133260.s003.doc]

**S3 Table. Chain-based evaluation of different feature-based predictors on** DB312 (RB264)

| Feature^a^ | Recall | Precision | F1 | ACC | MCC | AUC |
| --- | --- | --- | --- | --- | --- | --- |
| PSSM | 0.511 (0.431) | 0.517 (0.456) | 0.491 (0.417) | 0.857 (0.826) | 0.422 (0.309) | 0.829 (0.746) |
| PSSM+CS | 0.523 (0.456) | 0.539 (0.455) | 0.507 (0.430) | 0.863 (0.825) | 0.441 (0.319) | 0.841 (0.751) |
| PSSM+CS+PS | 0.536 (0.462) | 0.543 (0.471) | 0.514 (0.437) | 0.864 (0.828) | 0.450 (0.330) | 0.851 (0.758) |
| PSSM+CS+PS+PC | 0.542 (0.466) | 0.549 (0.477) | 0.520 (0.439) | 0.865 (0.829) | 0.456 (0.334) | 0.854 (0.760) |
| PSSM+CS+PS+PC+IP | 0.545 (0.468) | 0.549 (0.474) | 0.522 (0.442) | 0.866 (0.829) | 0.459 (0.336) | 0.855 (0.762) |
| PSSM+CS+PS+PC+IP+SP | 0.555 (0.469) | 0.545 (0.474) | 0.524 (0.443) | 0.865 (0.829) | 0.461 (0.337) | 0.855 (0.761) |
| PSSM+CS+PS+PC+IP+SP+GF | 0.579 (0.502) | 0.539 (0.474) | 0.532 (0.454) | 0.861 (0.823) | 0.466 (0.343) | 0.852 (0.760) |

^a^PSSM: position specific scoring matrix, CS: residue conservation scores, PS: predicted structural features, PC: physicochemical properties,

IP: interface propensity, SP: sequential position, and GF: global features.
