# Supplementary material for: SNBRFinder: A Sequence-Based Hybrid Algorithm for Enhanced Prediction of Nucleic Acid-Binding Residues
Source: PLoS One. 2015 Jul 15;10(7):e0133260. doi: 10.1371/journal.pone.0133260 (PMC4503397; doi:10.1371/journal.pone.0133260)
Supplement: S5 Table — (DOC) [file pone.0133260.s005.doc]

**S5 Table. Chain-based evaluation of different machine learning models and training sets on DB312 (RB264)**

| Training set | Classifier^a^ | Recall | Precision | F1 | ACC | MCC | AUC |
| --- | --- | --- | --- | --- | --- | --- | --- |
| Balanced | NB | 0.681 (0.535) | 0.308 (0.302) | 0.389 (0.352) | 0.698 (0.700) | 0.286 (0.176) | 0.721 (0.619) |
|  | NN | 0.518 (0.434) | 0.412 (0.382) | 0.440 (0.384) | 0.817 (0.792) | 0.345 (0.246) | 0.796 (0.703) |
|  | RF | 0.545 (0.500) | 0.461 (0.403) | 0.461 (0.387) | 0.826 (0.776) | 0.384 (0.261) | 0.808 (0.681) |
|  | SVM | 0.568 (0.486) | 0.504 (0.445) | 0.502 (0.433) | 0.847 (0.814) | 0.432 (0.318) | 0.840 (0.751) |
| Unbalanced | NB | 0.642 (0.550) | 0.317 (0.297) | 0.388 (0.353) | 0.718 (0.691) | 0.285 (0.171) | 0.717 (0.613) |
|  | NN | 0.487 (0.456) | 0.463 (0.396) | 0.451 (0.395) | 0.839 (0.799) | 0.369 (0.263) | 0.798 (0.700) |
|  | RF | 0.539 (0.482) | 0.458 (0.404) | 0.457 (0.387) | 0.829 (0.786) | 0.381 (0.262) | 0.795 (0.678) |
|  | SVM | 0.579 (0.502) | 0.539 (0.474) | 0.532 (0.454) | 0.861 (0.823) | 0.466 (0.343) | 0.852 (0.760) |

^a^NB: naive Bayes, NN: neural networks, RF: random forest, and SVM: support vector machines.
