# Supplementary material for: SNBRFinder: A Sequence-Based Hybrid Algorithm for Enhanced Prediction of Nucleic Acid-Binding Residues
Source: PLoS One. 2015 Jul 15;10(7):e0133260. doi: 10.1371/journal.pone.0133260 (PMC4503397; doi:10.1371/journal.pone.0133260)
Supplement: S6 Table — (DOC) [file pone.0133260.s006.doc]

**S6 Table. Chain**-based evaluation of our individual and hybrid predictors on DB312 (RB264)

| Group | NO. of chains | Method | Recall | Precision | F1 | ACC | MCC | AUC |
| --- | --- | --- | --- | --- | --- | --- | --- | --- |
| HHscore ≥ 85% | 216 (159) | SNBRFinder^F^ | 0.632 (0.532) | 0.573 (0.490) | 0.579 (0.484) | 0.873 (0.844) | 0.517 (0.384) | 0.874 (0.784) |
|  |  | SNBRFinder^T^ | 0.558 (0.449) | 0.650 (0.539) | 0.578 (0.469) | 0.894 (0.857) | 0.533 (0.396) | N/A (N/A) |
|  |  | SNBRFinder | 0.685 (0.568) | 0.606 (0.551) | 0.624 (0.535) | 0.887 (0.866) | 0.569 (0.448) | 0.894 (0.815) |
| HHscore < 85% | 96 (105) | SNBRFinder^F^ | 0.462 (0.456) | 0.463 (0.450) | 0.425 (0.409) | 0.836 (0.791) | 0.351 (0.282) | 0.797 (0.718) |
|  |  | SNBRFinder^T^ | 0.084 (0.042) | 0.234 (0.139) | 0.113 (0.057) | 0.849 (0.756) | 0.090 (0.004) | N/A (N/A) |
|  |  | SNBRFinder | 0.477 (0.456) | 0.459 (0.450) | 0.429 (0.409) | 0.833 (0.791) | 0.354 (0.282) | 0.797 (0.718) |
| All | 312 (264) | SNBRFinder^F^ | 0.579 (0.502) | 0.539 (0.474) | 0.532 (0.454) | 0.861 (0.823) | 0.466 (0.343) | 0.852 (0.760) |
|  |  | SNBRFinder^T^ | 0.412 (0.287) | 0.522 (0.379) | 0.435 (0.305) | 0.881 (0.817) | 0.397 (0.240) | N/A (N/A) |
|  |  | SNBRFinder | 0.621 (0.523) | 0.561 (0.511) | 0.564 (0.484) | 0.871 (0.836) | 0.503 (0.382) | 0.869 (0.780) |
