# Supplementary material for: SNBRFinder: A Sequence-Based Hybrid Algorithm for Enhanced Prediction of Nucleic Acid-Binding Residues
Source: PLoS One. 2015 Jul 15;10(7):e0133260. doi: 10.1371/journal.pone.0133260 (PMC4503397; doi:10.1371/journal.pone.0133260)
Supplement: S7 Table — (DOC) [file pone.0133260.s007.doc]

**S7 Table. Chain**-based evaluation of our proposed predictors on various datasets

| Dataset | Method | Recall | Precision | F1 | ACC | MCC | AUC |
| --- | --- | --- | --- | --- | --- | --- | --- |
| DB123 | PSSM | 0.504 | 0.460 | 0.450 | 0.819 | 0.361 | 0.799 |
|  | SNBRFinder^F^ | 0.570 | 0.483 | 0.490 | 0.827 | 0.409 | 0.821 |
|  | SNBRFinder^T^ | 0.262 | 0.385 | 0.293 | 0.857 | 0.253 | N/A |
|  | SNBRFinder | 0.602 | 0.493 | 0.512 | 0.834 | 0.435 | 0.833 |
| DB232 | PSSM | 0.517 | 0.345 | 0.385 | 0.851 | 0.329 | 0.805 |
|  | SNBRFinder^F^ | 0.557 | 0.373 | 0.417 | 0.861 | 0.368 | 0.834 |
|  | SNBRFinder^T^ | 0.269 | 0.372 | 0.284 | 0.900 | 0.259 | N/A |
|  | SNBRFinder | 0.579 | 0.379 | 0.430 | 0.865 | 0.384 | 0.845 |
| DB374 | PSSM | 0.395 | 0.472 | 0.402 | 0.915 | 0.375 | 0.755 |
|  | SNBRFinder^F^ | 0.486 | 0.470 | 0.452 | 0.910 | 0.420 | 0.774 |
|  | SNBRFinder^T^ | 0.356 | 0.398 | 0.354 | 0.912 | 0.328 | N/A |
|  | SNBRFinder | 0.491 | 0.485 | 0.463 | 0.917 | 0.433 | 0.780 |
| RB106 | PSSM | 0.484 | 0.480 | 0.457 | 0.788 | 0.317 | 0.740 |
|  | SNBRFinder^F^ | 0.529 | 0.487 | 0.479 | 0.789 | 0.340 | 0.759 |
|  | SNBRFinder^T^ | 0.194 | 0.336 | 0.216 | 0.758 | 0.164 | N/A |
|  | SNBRFinder | 0.577 | 0.519 | 0.521 | 0.796 | 0.382 | 0.772 |
| RB144 | PSSM | 0.475 | 0.474 | 0.447 | 0.782 | 0.295 | 0.736 |
|  | SNBRFinder^F^ | 0.546 | 0.486 | 0.485 | 0.787 | 0.336 | 0.755 |
|  | SNBRFinder^T^ | 0.217 | 0.358 | 0.247 | 0.758 | 0.178 | N/A |
|  | SNBRFinder | 0.602 | 0.508 | 0.525 | 0.795 | 0.379 | 0.772 |
| RB198 | PSSM | 0.429 | 0.444 | 0.414 | 0.806 | 0.287 | 0.727 |
|  | SNBRFinder^F^ | 0.474 | 0.461 | 0.440 | 0.807 | 0.315 | 0.741 |
|  | SNBRFinder^T^ | 0.230 | 0.360 | 0.258 | 0.795 | 0.198 | N/A |
|  | SNBRFinder | 0.505 | 0.490 | 0.471 | 0.818 | 0.353 | 0.761 |
