# Supplementary material for: SNBRFinder: A Sequence-Based Hybrid Algorithm for Enhanced Prediction of Nucleic Acid-Binding Residues
Source: PLoS One. 2015 Jul 15;10(7):e0133260. doi: 10.1371/journal.pone.0133260 (PMC4503397; doi:10.1371/journal.pone.0133260)
Supplement: S8 Table — (DOC) [file pone.0133260.s008.doc]

**S8 Table. Chain**-based evaluation of other sequence- and structure-based predictors on DB312 (RB264)

| Type | Method^a^ | Recall | Precision | F1 | ACC | MCC | AUC |
| --- | --- | --- | --- | --- | --- | --- | --- |
| Template | BLAST | 0.227 (0.198) | 0.360 (0.356) | 0.255 (0.226) | 0.858 (0.810) | 0.226 (0.184) | N/A (N/A) |
|  | TMalign | 0.414 (0.274) | 0.529 (0.388) | 0.443 (0.293) | 0.876 (0.795) | 0.395 (0.222) | N/A (N/A) |
|  | SPalign | 0.429 (0.333) | 0.547 (0.440) | 0.458 (0.350) | 0.878 (0.816) | 0.412 (0.277) | N/A (N/A) |
| Feature | NBRFeature^SEQ^ | 0.606 (0.558) | 0.552 (0.487) | 0.554 (0.485) | 0.867 (0.821) | 0.491 (0.375) | 0.863 (0.780) |
|  | NBRFeature^STR^ | 0.612 (0.508) | 0.480 (0.463) | 0.511 (0.446) | 0.836 (0.813) | 0.436 (0.332) | 0.840 (0.757) |
|  | NBRFeature | 0.625 (0.547) | 0.560 (0.513) | 0.565 (0.485) | 0.868 (0.827) | 0.504 (0.383) | 0.871 (0.782) |
| Hybrid | SNBRFinder^BLAST^ | 0.576 (0.503) | 0.545 (0.487) | 0.532 (0.462) | 0.863 (0.827) | 0.468 (0.354) | 0.853 (0.763) |
|  | NBRDetector^TMalign^ | 0.674 (0.552) | 0.570 (0.530) | 0.595 (0.505) | 0.875 (0.839) | 0.537 (0.407) | 0.885 (0.798) |
|  | NBRDetector | 0.679 (0.561) | 0.572 (0.532) | 0.598 (0.518) | 0.876 (0.844) | 0.540 (0.421) | 0.887 (0.804) |

^a^NBRFeature: a structure-based feature predictor in our previous work, NBRDetector: the combination of NBRFeature and SPalign, NBRDetector^TMalign^:

the combination of NBRFeature and TMalign, and SNBRFinder^BLAST^: the combination of SNBRFinder^F^ and BLAST.
