# Supplementary material for: SNBRFinder: A Sequence-Based Hybrid Algorithm for Enhanced Prediction of Nucleic Acid-Binding Residues
Source: PLoS One. 2015 Jul 15;10(7):e0133260. doi: 10.1371/journal.pone.0133260 (PMC4503397; doi:10.1371/journal.pone.0133260)
Supplement: S10 Table — (DOC) [file pone.0133260.s010.doc]

**S10 Table. Chain-based evaluation of sequence- and structural model-based predictions on DB35** (RB36)

| Type^a^ | Method | Recall | Precision | F1 | ACC | MCC | AUC |
| --- | --- | --- | --- | --- | --- | --- | --- |
| Structure | NBRFeature | 0.642 (0.632) | 0.570 (0.523) | 0.576 (0.553) | 0.858 (0.774) | 0.507 (0.401) | 0.873 (0.779) |
|  | NBRTemplate^b^ | 0.390 (0.304) | 0.522 (0.470) | 0.409 (0.341) | 0.849 (0.774) | 0.356 (0.259) | N/A (N/A) |
|  | NBRDetector | 0.691 (0.638) | 0.581 (0.562) | 0.605 (0.576) | 0.862 (0.797) | 0.539 (0.444) | 0.880 (0.801) |
| Model | NBRFeature | 0.601 (0.649) | 0.531 (0.493) | 0.535 (0.537) | 0.847 (0.760) | 0.461 (0.381) | 0.843 (0.765) |
|  | NBRTemplate | 0.359 (0.293) | 0.476 (0.464) | 0.379 (0.334) | 0.847 (0.766) | 0.325 (0.241) | N/A (N/A) |
|  | NBRDetector | 0.648 (0.647) | 0.537 (0.526) | 0.563 (0.561) | 0.850 (0.782) | 0.489 (0.416) | 0.854 (0.784) |
| Sequence | SNBRFinder^F^ | 0.620 (0.602) | 0.538 (0.502) | 0.550 (0.532) | 0.854 (0.780) | 0.480 (0.381) | 0.852 (0.762) |
|  | SNBRFinder^T^ | 0.387 (0.266) | 0.456 (0.311) | 0.396 (0.277) | 0.857 (0.752) | 0.352 (0.183) | N/A (N/A) |
|  | SNBRFinder | 0.658 (0.611) | 0.565 (0.521) | 0.586 (0.548) | 0.861 (0.789) | 0.517 (0.403) | 0.866 (0.780) |

^a^Structure: native structure, Model: structural model, and Sequence: protein sequence.

^b^NBRTemplate: the template predictor implemented with SPalign.
