# Supplementary material for: SNBRFinder: A Sequence-Based Hybrid Algorithm for Enhanced Prediction of Nucleic Acid-Binding Residues
Source: PLoS One. 2015 Jul 15;10(7):e0133260. doi: 10.1371/journal.pone.0133260 (PMC4503397; doi:10.1371/journal.pone.0133260)
Supplement: S12 Table — (DOC) [file pone.0133260.s012.doc]

**S12 Table. Comparison of SNBFinder and RNABindRPlus on** RB44

| Evaluation | Method | Recall | Precision | F1 | ACC | MCC | AUC |
| --- | --- | --- | --- | --- | --- | --- | --- |
| Residue-based | SNBRFinder^F^ | 0.711 | 0.462 | 0.560 | 0.779 | 0.439 | 0.827 |
|  | SVMOpt | 0.791 | 0.416 | 0.545 | 0.739 | 0.425 | 0.825 |
|  | SNBRFinder^T^ | 0.311 | 0.541 | 0.395 | 0.811 | 0.308 | N/A |
|  | HomPRIP | 0.285 | 0.564 | 0.379 | 0.815 | 0.306 | N/A |
|  | SNBRFinder | 0.721 | 0.501 | 0.591 | 0.802 | 0.479 | 0.843 |
|  | RNABindRPlus | 0.696 | 0.528 | 0.600 | 0.816 | 0.492 | 0.840 |
| Chain-based | SNBRFinder^F^ | 0.661 | 0.429 | 0.509 | 0.764 | 0.369 | 0.774 |
|  | SVMOpt | 0.744 | 0.394 | 0.500 | 0.725 | 0.354 | 0.767 |
|  | SNBRFinder^T^ | 0.295 | 0.351 | 0.304 | 0.790 | 0.219 | N/A |
|  | HomPRIP | 0.265 | 0.257 | 0.253 | 0.794 | 0.197 | N/A |
|  | SNBRFinder | 0.686 | 0.469 | 0.546 | 0.784 | 0.414 | 0.798 |
|  | RNABindRPlus | 0.639 | 0.482 | 0.531 | 0.800 | 0.406 | 0.782 |
